# Effectiveness of a Nurse-Managed Protocol to Prevent Hypoglycemia in Hospitalized Patients with Diabetes

**DOI:** 10.1155/2015/173956

**Published:** 2015-04-16

**Authors:** Giuseppe Marelli, Fausto Avanzini, Giuseppe Iacuitti, Enrico Planca, Ilaria Frigerio, Giovanna Busi, Liliana Carlino, Laura Cortesi, Maria Carla Roncaglioni, Emma Riva

**Affiliations:** ^1^Diabetes and Metabolic Diseases Unit, Ospedale di Desio, Desio, Italy; ^2^Division of Cardiology and Intensive Cardiac Care Unit, Ospedale di Desio, Desio, Italy; ^3^IRCCS-Istituto di Ricerche Farmacologiche “Mario Negri”, Via Giuseppe La Masa 19, 20156 Milan, Italy

## Abstract

*Background*. Hypoglycemia due to inadequate carbohydrate intake is a frequent complication of insulin treatment of diabetic in-patients. *Objective.* To assess the effectiveness of a nurse-managed protocol to prevent hypoglycemia during subcutaneous insulin treatment. *Design*. Prospective pre-post-intervention study. *Methods*. In 350 consecutive diabetic in-patients the incidence of hypoglycemia (blood glucose < 70 mg/dL) during subcutaneous insulin treatment was assessed before (phase A) and after (phase B) the protocol was adopted to permit (1) the patient to opt for substitutive food to integrate incomplete carbohydrate intake in the meal; (2) in case of lack of appetite or repeatedly partial intake of the planned food, prandial insulin administered at the end of the meal to be related to the actual amount of carbohydrates eaten; (3) intravenous infusion of glucose during prolonged fasting. *Results*. Eighty-four patients in phase A and 266 in phase B received subcutaneous insulin for median periods of, respectively, 7 (Q1–Q3 6–12) and 6 days (Q1–Q3 4–9). Hypoglycemic events declined significantly from 0.34 ± 0.33 per day in phase A to 0.19 ± 0.30 in phase B (*P* > 0.001). *Conclusions*. A nurse-managed protocol focusing on carbohydrate intake reduced the incidence of hypoglycemia in patients with diabetes receiving subcutaneous insulin in hospital.

## 1. Introduction

Hyperglycemia is common in patients with diabetes in hospital and several guidelines have suggested using basal and prandial subcutaneous (*s.c.*) insulin in non-critically ill patients as first-choice pharmacological treatment to ensure the best possible glycemic control, combined with an appropriate nutrition scheme [1–5]. However, intensive treatment of hyperglycemia risks raising the rate of hypoglycemia and this may worsen the patient's prognosis [6–9].

No or incomplete carbohydrate intake in the prescribed meal plan exposes patients on* s.c.* insulin to hypoglycemic events [6, 10, 11]. Skipping one or more meals often happens in hospital to comply with fasting conditions for certain instrumental and clinical procedures. Organizational problems often cause delays in eating the meal set down in the dietary plan. In addition, the patients' clinical conditions and their drugs may cause lack of appetite, nausea, or vomiting, all leading to reduced food intake. A similar situation can arise as the consequence of scarce palatability or unfamiliarity of the food served in hospital.

Since in our department we frequently observed hypoglycemic episodes after incomplete meals or during prolonged fasting in patients with diabetes treated with* s.c.* insulin, we introduced a simple nurse-managed protocol aimed at ensuring the recommended carbohydrate intake every day in these patients and/or reducing the dosage of prandial insulin accordingly, under the hypothesis that this might prevent hypoglycemic events and/or reduce their severity.

## 2. Patients, Material, and Methods

Since May 2006 all patients with diabetes or hyperglycemia (BG >200 mg/dL) admitted to our Cardiology Department for suspected acute coronary syndrome (ACS) have been treated according to the Desio Diabetes Diagram (DDD) protocol we designed based on current guidelines [12–14]. During the acute phase in the intensive cardiac care unit (ICCU) patients were treated with an insulin infusion protocol (IIP) combined with parenteral nutrition using intravenous (*i.v.*) glucose solution [15] and subsequently in the cardiology ward with* s.c.* insulin, according to the basal-bolus-correction scheme, associated with oral nutrition [16]. The initial daily dose of* s.c.* insulin was double the amount given in the last 12 hours of infusion, as follows:

- half the daily amount of insulin was given as a single dose, usually at lunchtime, as basal insulin (glargine);
- the other half was given as prandial insulin (lispro) just before the three main meals and the amount was proportional to the carbohydrates in the planned diet: 25% at breakfast, 40% at lunch, and 35% at dinner.

Each patient received a diet based on height and sex, containing a fixed amount of carbohydrates equivalent to 55–60% of the total calories calculated on the base of 22 kcal/day per kg of the ideal body weight.

At the end of the first year of application of the DDD protocol we evaluated its efficacy and safety. Because of the high frequency of hypoglycemic episodes, often associated with fasting or incomplete carbohydrate intake, in 2007 we started a specific nurse-managed protocol aimed at preventing hypoglycemia focusing primarily on ensuring each patient receiving subcutaneous insulin had the recommended daily carbohydrate intake, as follows (Table 4):

1. if examinations or procedures requiring prolonged fasting are scheduled, a glucose solution must be infused* i.v.* from the first skipped meal until oral feeding is reestablished, stopping prandial insulin and maintaining the basal insulin;
2. at the end of each meal, nurses must make sure the patient has eaten the planned amount of carbohydrates. To help the nurse check the patient's carbohydrate intake, we devised a new set of diets in which only few, easily identified foods contained a fixed amount of carbohydrates: at breakfast, the foods containing carbohydrates were milk and bread, at lunch pasta or rice, bread, and fruit and at dinner soup, bread, and fruit. If the patient has eaten only part or none of the planned carbohydrates, the nurse suggests s/he should make up for the uneaten amount, offering other foods easily available in the ward containing known amounts of carbohydrates;
3. in case of lack of appetite, or repeated partial intake of the planned food, prandial insulin must be given at the end of the meal, the dose matching the amount of carbohydrates actually eaten.

To explain the protocol for preventing hypoglycemia to the medical and nursing staff and to train nurses in its use, we organized brief meetings to illustrate the composition of the diet of patients with diabetes, to identify the foods containing carbohydrates and to explain how to replace uneaten or partially eaten food with some substitute food containing a similar amount of carbohydrates.

In this prospective pre-post-intervention study all patients with diabetes or hyperglycemia consecutively admitted to our cardiology department for suspected ACS were prospectively monitored for hypoglycemic events during their hospital stay in the year before the implementation of the protocol and the next three years.

Blood glucose (BG) was measured at the bedside seven times every day throughout* s.c.* insulin treatment: before and 1.5–2.0 hours after breakfast, lunch, and dinner, and at bedtime. BG was also measured any time the patient experienced symptoms suggesting hypoglycemia.

During phase A and phase B of the study the glycemic goals did not change: the glycemic target during the acute phase of* i.v.* insulin infusion was 100–139 mg/dL and during the subacute phase with* s.c.* insulin, preprandial and postprandial targets were respectively less than 120 and 160 mg/dL, according to European recommendations in vigour when the study was done [12].

In 2008 to assess the satisfaction of nurses after the DDD protocol had been implemented for more than one year, all the nurses of the ICCU and cardiology ward were asked to complete a questionnaire anonymously. Each answer was rated between 0 (low/little) and 6 (high/much) and scores ≥4 were considered for the analysis.

## 3. Statistical Analysis

We used the chi-square test to analyze proportions,* t*-test for means, and Wilcoxon test for median analyses. To assess the efficacy of the protocol for preventing hypoglycemic events during* s.c.* insulin treatment, we analyzed all hypoglycemic episodes in the year before (phase A) and during the first three years of implementation of the protocol (phase B). A hypoglycemic event was defined as capillary BG <70 mg/dL with or without related symptoms; a severe hypoglycemic event was defined as BG <40 mg/dL. The incidence of hypoglycemic events during* s.c.* insulin treatment is expressed as follows: (i) the proportion of patients having one or more hypoglycemic episodes; (ii) the rate of hypoglycemic events per day of insulin treatment; and (iii) the mean number of hypoglycemic events per patient [17]. Since the hospital stay for patients admitted with ACS slightly declined during the study, we took the rate of hypoglycemic episodes per day as the primary end point.

We also constructed Poisson regression to estimate the incidence rate ratio and multivariable logistic models to estimate the odds ratio for the risk of hypoglycemic events considering known confounding variables [6, 11, 18] and implementation of the protocol (phase B). The analyses considered continuous variables (age, body mass index, estimated glomerular filtration rate, initial daily* s.c.* insulin dose, and length of treatment) and categorical variables (sex, history of diabetes and malignancy, liver failure, infections during hospital stay, and implementation of the protocol). All *P* values were two-tailed and considered significant if <0.05. All statistical analyses were performed by Stata 12.0 (StataCorp, College Station, TX, USA) software.

## 4. Results

A total of 422 patients with diabetes or hyperglycemia (BG >200 mg/dL) at admission to our ICCU for suspected ACS were included. We excluded 19 patients not completing* i.v.* insulin infusion, 47 not transiting from* i.v.* to* s.c.* insulin treatment, and six who stopped the protocol the first day of* s.c.* insulin (four refused to continue* s.c.* insulin and two died).

The study population therefore comprised 350 patients: 84 treated with* s.c.* insulin before (phase A) and 266 during (phase B) the implementation of the protocol.  Table 1 shows the main characteristics of the two groups, which were similar, except for BMI, which was higher in patients of phase A than in phase B (*P* < 0.001). The mean duration of* s.c.* insulin treatment was seven days (Q1–Q3 6–12) in phase A and six (Q1–Q3 4–9) in phase B (*P* = 0.002).

During the study there were 639 episodes of hypoglycemia (284 during phase A and 355 during phase B) and 29 episodes of severe hypoglycemia (20 in phase A and nine in phase B). During the implementation of the protocol, the rate of hypoglycemic episodes per day of* s.c.* insulin treatment decreased significantly (*P* < 0.001) from 0.34 ± 0.33 (phase A) to 0.19 ± 0.30 (phase B) (Table 2). The frequency of hypoglycemic episodes per day of* s.c.* insulin treatment was similar in the first, second, and third years of implementation of the protocol (Phase B), respectively, 0.20 ± 0.24, 0.18 ± 0.39, and 0.19 ± 0.24 (*P* = 0.431). Table 2 also shows the proportions of patients with at least one hypoglycemic episode during* s.c.* insulin in both phases, as well as the mean number of hypoglycemic events per patient. All these variables were significantly lower during phase B and again, no significant differences were observed between years in phase B.

The rate of severe hypoglycemic events per day of* s.c.* insulin treatment dropped significantly from phase A to phase B from 0.016 ± 0.045 to 0.004 ± 0.028 (*P* = 0.011) (Table 2). The frequency of severe hypoglycemic episodes per day of* s.c.* insulin treatment was similar in the first, second, and third years of implementation of the protocol (Phase B), respectively, 0.002 ± 0.140, 0.009 ± 0.045, and 0.001 ± 0.001 (*P* = 0.437). Table 2 also reports the proportion of patients with BG <40 mg/dL during* s.c.* insulin in both phases and the mean number of severe hypoglycemic events per patient. All these variables were significantly lower during phase B and no significant differences were observed between years in phase B.

The significant reduction of all and severe hypoglycemic events in phase B was maintained in the multivariable analyses, after adjusting for known confounders (Table 3).

In 2008 29 nurses out of 30 agreed to complete the questionnaire on satisfaction with the DDD protocol. Difficulties in training for the use of the protocol were reported by 34.5%; 62.1% considered the protocol easy to use. An increased work burden was reported by 93.1%. However, the nursing personnel found the protocol very helpful in improving the control of hyperglycemia and the quality of care of diabetic inpatients (resp., 96.4% and 89.3%). Almost all nurses felt that the protocol had made them more independent (84.6%). Overall satisfaction with the protocol was expressed by 85.7% of nurses.

## 5. Discussion

Tight glycemic control in diabetic in-patients is important to reduce complications associated with hyperglycemia [19–23]. However, one of the major obstacles is the fear that too tight control may cause hypoglycemia [24]. Hypoglycemia too is a negative prognostic factor during hospital stay [6–9]. Therefore, interventions aimed at preventing hypoglycemia are an essential part of in-patient diabetes management.

Multiple risk factors for hypoglycemia may coexist in hospital and some can in fact be easily managed. The reduction of carbohydrates intake frequently triggers hypoglycemia [6, 10, 11]. The patients' clinical and psychological status, and often the poor palatability of the food served in hospital can reduce their appetite, causing inadequate food intake, particularly of carbohydrates. Another frequent cause of reduced carbohydrates intake is the fasting required for some investigations or procedures. Though many in-patient episodes of hypoglycemia are preventable, clinical guidelines suggest protocols for its treatment rather than prevention, so hospital nurses and physicians pay more attention to treating than preventing it [1–5].

After having observed frequent hypoglycemic episodes, on average, one every three days, in our patients treated with* s.c.* insulin according to the basal-bolus correction scheme, as suggested in the literature, we drew up nurse-managed procedures aimed at preventing hypoglycemia (Table 4). These interventions nearly halved the hypoglycemic events and, more importantly, the frequency of severe hypoglycemic events was reduced fourfold.

### 5.1. Comparison with the Literature

We documented a high incidence of hypoglycemic events: over half the patients treated with* s.c.* insulin according to the basal-bolus-correction scheme had at least one such episode. Other authors have assessed the incidence of hypoglycemic events during intensive* s.c.* insulin in hospital and the frequencies reported vary widely, ranging from less than 10% to 50% [10, 25–30]. These results are not easily comparable with ours because of the different criteria used to define hypoglycemia, the timing of BG measurements, the type of treatments, the glycemic target, and the length of hospital stay. The high incidence of hypoglycemia in our population is very likely due to the aggressive glycemic targets adopted in the study years, lower than those now recommended [10–14]. A further reason may lie in our frequent daily measurements of BG (before and after each meal and at bedtime) in all patients treated with* s.c.* insulin, which detect even mild hypoglycemic events that are generally asymptomatic.

There are no guidelines suggesting specific strategies to prevent hypoglycemia in hospital caused by insufficient carbohydrate intake with the diet or prolonged fasting [1–5]. This is one of the few studies assessing the efficacy of feasible, practical nurse-managed strategies to reduce hypoglycemic events [31] focusing on ensuring recommended carbohydrate intake every day and/or reducing the dosage of prandial insulin accordingly.

### 5.2. Nurses' Satisfaction with the Protocol

In relation to the feasibility of the protocol, only one-third of nurses mentioned difficulties in training. The increase in their work burden reported by the majority of nurses was mainly due to the time required for the seven daily BG measurements, before and after meals and at bedtime, and for the review of all meals consumed. Although the protocol has added some work for the nursing staff, it met with their favor in virtue of the immediate results in terms of its efficacy in controlling hyperglycemia and preventing both mild and severe hypoglycemic episodes. Moreover, nurses particularly appreciated the gain in autonomy and involvement in the management of diabetic patients.

### 5.3. Strengths of the Study

Our study used a novel approach to the problem of hypoglycemia in hospitalized patients, since it focused on prevention rather than treatment of the hypoglycemic events, by the application of a simple nurse-managed protocol. The protocol was accepted well by nurses working in our cardiology department and easily applied in all diabetic in-patients treated with* s.c.* insulin. The characteristics of the protocol make its application exportable to other clinical contexts more or less specialized in the cure of diabetic patients.

The similar frequencies of hypoglycemic events over the three years of the study suggest the rapid and effective training for nurses from the first period of implementation of the protocol.

### 5.4. Limits of the Study

This was a prospective pre-post-intervention study in a single center. By definition, the results of pre-post-intervention studies cannot be considered to demonstrate a direct causal relationship between the strategies adopted and the findings. However, this study did document the effectiveness of our protocol. The main differences in hypoglycemia incidences between phase A and phase B can hardly be ascribed to any reason other than the nurse protocol. In fact, with the exception of BMI, the characteristics of patients were well matched in the two phases of the study (Table 1). The multivariable analyses, which included most of the confounding factors, support the conclusion that the lower frequency of hypoglycemic events in phase B was due to the protocol application (Table 3).

We are unable to separate the role of the three specific interventions in the protocol (Table 4) because we did not record how many times each one was applied. Taking into account the frequencies of the three conditions that put patients at risk of hypoglycemia, we believe each intervention was useful. The reduction of the hypoglycemic episodes could also at least partly reflect the training to make the medical and nursing teams aware of the need for more careful therapeutic regimens, and for “educating” patients about their diet, particularly the importance of eating the full amount of carbohydrates contained in the food provided. These components are anyway part of our protocol.

## 6. Conclusion

This study shows that simple nurse-managed preventive measures significantly reduced hypoglycemia in diabetic hospitalized patients treated with* s.c.* insulin. Considering that hypoglycemic events are frequent in the hospital setting and have negative prognostic implications, close attention is needed to ensure patients have* i.v.* glucose infused during prolonged fasting, consume the planned amount of carbohydrates with the diet, and, in case of lack of appetite or repeated reduced intake of planned carbohydrates, are given a corresponding lower dosage of prandial insulin at the end of the meal. This should become an in-hospital routine preventive strategy with insulin treatment.

## Figures and Tables

**Table 1:** Patients' main characteristics.

	Phase A (84 patients)	Phase B (266 patients)	*P*
Age (years) mean ± SD	70.2 ± 10.1	70.9 ± 11.0	0.396
<65 (no., %)	21 (25.0)	69 (25.9)	0.173
65–74 (no., %)	34 (40.5)	80 (30.1)	
≥75 (no., %)	29 (34.5)	117 (44.0)	
Male (number, %)	60 (71.4)	168 (63.2)	0.166
History of diabetes (number, %)			
(i) None	4 (4.8)	18 (6.7)	0.076
(ii) Type 1	3 (3.5)	12 (4.5)	
(iii) Type 2	75 (89.3)	236 (88.8)	
(iv) Other types	2 (2.4)	0 (0.0)	
Glucose-lowering drugs used at home (number, %)			
(i) None	7 (8.3)	29 (10.9)	0.499
(ii) Oral	53 (63.1)	149 (56.0)	
(iii) Insulin	22 (26.2)	74 (27.8)	
(iv) Insulin and oral drugs	2 (2.4)	14 (5.3)	
History of malignant cancer (number, %)	9 (10.7)	31 (12.0)	0.805
Body mass index (kg/m^2^)			
<18 (number, %)	0 (0.0)	2 (0.8)	<0.001
18–24 (number, %)	18 (21.4)	87 (32.7)	
25–29 (number, %)	39 (46.4)	118 (44.4)	
≥30 (number, %)	27 (32.1)	58 (21.8)	
Blood glucose at admission (mg/dL)			
<200 (number, %)	53 (63.1)	174 (65.4)	0.895
≥200 (number, %)	31 (36.9)	91 (34.2)	
HbA1c at admission (mmol/mol, %)			
<6 (no., %)	4 (4.8)	33 (12.4)	0.989
6–6.9 (no., %)	31 (36.9)	83 (31.2)	
7–7.9 (no., %)	25 (29.8)	54 (20.3)	
≥8 (no., %)	24 (28.6)	92 (35.6)	
eGFR at hospital admission (mL/min/1.73 m^2^)			
≥90	17 (20.2)	50 (18.8)	0.944
60–89	27 (32.1)	95 (35.7)	
30–59	30 (35.7)	92 (34.6)	
<30	10 (11.9)	29 (10.9)	
Liver failure (number, %)	2 (2.4)	5 (2.3)	0.778
Infection in hospital (number, %)	17 (20.2)	59 (22.2)	0.707
Initial *s.c.* insulin dose (IU/kg/day)			
<0.6	57 (67.9)	200 (75.2)	0.168
≥0.6	27 (32.1)	65 (24.4)	
Diagnosis at discharge (number, %)			
(i) STEMI	21 (25.0)	94 (35.3)	0.0760
(ii) NSTEMI	44 (52.4)	111 (41.7)	
(iii) Unstable angina	3 (3.6)	23 (8.7)	
(iv) Other	16 (19.0)	38 (14.3)	

SD = standard deviation; STEMI = ST-segment elevation acute myocardial infarction; NSTEMI = non-ST-segment elevation acute myocardial infarction; eGFR = estimated glomerular filtration rate calculated according to CKD-EPI formula.

**Table 2:** All hypoglycemic (blood glucose <70 mg/dL) and severe episodes (blood glucose <40 mg/dL) during phase A and phase B.

Variable	Phase A (84 patients)	Phase B (266 patients)	*P*
Number of all hypoglycemic events per day (mean ± SD)	0.34 ± 0.33	0.19 ± 0.30	<0.001
Patients with any hypoglycemic events (number, %)	61 (72.6)	144 (54.1)	0.003
Number of all hypoglycemic events per patient (mean ± SD)	3.4 ± 4.1	1.3 ± 2.1	<0.001

Numbers of severe hypoglycemic events per day (mean ± SD)	0.016 ± 0.045	0.004 ± 0.028	0.011
Patients with severe hypoglycemic events (number, %)	14 (16.7)	7 (0.3)	<0.001
Number of severe hypoglycemic events per patient (mean ± SD)	0.24 ± 0.65	0.03 ± 0.23	0.003

**Table 3:** Multivariable analyses: adjusted ratio of the protocol implementation (phase B) for all and severe hypoglycemic events.

Multivariable model	Variable	Adjusted ratio^∗^ (95% CI)	*P*
1	Mean number of all hypoglycemic events per day with the protocol implementation	0.549 (0.466–0.646)	<0.001
2	Occurrence of any hypoglycemic event during hospital stay with the protocol implementation	0.497 (0.276–0.893)	0.019
3	Mean number of all hypoglycemic events per patient with the protocol implementation	0.100 (0.085–0.120)	<0.001

4	Mean number of severe hypoglycemic events per day with the protocol implementation	0.258 (0.114–0.582)	0.001
5	Severe hypoglycemic event during hospital stay with the protocol implementation	0.152 (0.045–0.519)	0.003
6	Mean number of severe hypoglycemic events per patient with the protocol implementation	0.048 (0.019–0.119)	<0.001

^∗^Incidence rate ratio in models 1, 3, 4, and 6; odds ratio in models 2 and 5. CI = confidence intervals.

All models included as covariates: age, sex, history of diabetes, history of malignancy, liver failure, body mass index (kg/m^2^), estimated glomerular filtration rate (mL/min/1.73 m^2^), initial daily *s.c.* insulin dose (IU/kg/day), infection during hospital stay, and protocol implementation (phase B). Models 2, 3, 5, and 6 also included as covariate the length of *s.c.* insulin treatment (days).

**Table:** (a) Make sure patients eat the expected amounts of carbohydrates in each meal and if they do not eat the whole carbohydrate ration, propose substitutive food with similar carbohydrate content. The following table gives some examples of food containing carbohydrates, easily found in the ward.

Carbohydrates	Type of food	Amount
10 g	Rusks	10 g (2 rusks)
	Breadsticks	15 g (4 breadsticks)
	Yogurt	250 g (2 jars)
	Milk	200 g (1 cup)

**Table:** (b) If the patient has no appetite or repeatedly eats only part of the food, give prandial insulin at the end of meal in relation to the amount of carbohydrates eaten, according to the following table.

Carbohydrate intake with meal	Prandial insulin to be given at the end of the meal
~100%	Prescribed dose
~50%	Half the prescribed dose
~0%	None

**Table:** (c) If examinations or procedures requiring fasting are planned, do not give prandial insulin at the missed/skipped meals but start the infusion of a glucose solution^∗^ following the following table until the patient can resume oral feeding.

Patient's sex and height	20% glucose solution in a central vein	10% glucose solution in a peripheral vein of good caliber	5% glucose solution in a peripheral vein of small caliber
F ≤160 cm	16 mL/h	32 mL/h	64 mL/h
F >160 cm M ≤175 cm	17 mL/h	35 mL/h	70 mL/h
M >175 cm	19 mL/h	39 mL/h	78 mL/h

^∗^The hourly infusion speed of glucose solution is calibrated to provide in 24 hours half the daily carbohydrate requirement set by the diet.
